# Spina Ventosa of the Left Index Finger in an Indian Girl With No Pulmonary Involvement: A Rare Case

**DOI:** 10.7759/cureus.44400

**Published:** 2023-08-30

**Authors:** Sankalp Yadav, Gautam Rawal, Madhan Jeyaraman

**Affiliations:** 1 Department of Medicine, Shri Madan Lal Khurana Chest Clinic, Moti Nagar, New Delhi, IND; 2 Department of Respiratory Medicine and Critical Care, Max Super Speciality Hospital, New Delhi, IND; 3 Department of Orthopedics, A.C.S. Medical College and Hospital, Dr. M.G.R. Educational and Research Institute, Chennai, IND

**Keywords:** biopsy, cbnaat/ xpert/ rif assay, tuberculous dactylitis, spina ventosa, mtb (mycobacterium tuberculosis)

## Abstract

*Mycobacterium tuberculosis* is a significant issue in endemic countries. The most common manifestation of skeletal tuberculosis in children is spondylitis, also known as Pott disease, but it may rarely involve small bones. Usually, a pulmonary focus is present from where the bacteria reach the extremities, but an isolated tuberculous involvement of the left index finger in a child without any pulmonary seeding is rare. It is a challenging diagnosis due to a lack of awareness among primary care physicians, the paucibacillary nature of the disease, and overlapping clinical features with other musculoskeletal disorders. A 13-year-old girl was brought in with complaints of pain, swelling, and discharging sinuses from her left index finger. A diagnosis was achieved after a histopathological correlation of clinical and radiological findings. She was started on anti-tubercular treatment for 12 months.

## Introduction

Tuberculosis is an infectious disease due to *Mycobacterium tuberculosis* from the family *Mycobacteriaceae* order Actinomycetales [1]. It has been known since ancient times that it is a significant cause of morbidity and mortality [2]. The disease could present in pulmonary or extrapulmonary forms which constitute 10-15% of all tuberculosis cases [2].

In 1-5% of children with untreated primary pulmonary tuberculosis, dissemination to the skeletal system during the first infection via the lymphohematogenous route results in bone and joint tuberculosis [3]. Within one to three years of the initial infection, symptoms of the skeletal infection appear [4]. On the contrary, adult skeletal involvement mainly occurs after the reactivation of the tuberculosis.

The most commonly affected musculoskeletal sites of tuberculosis are the spine (50-70%) (thoracic 50% > cervical 25% > lumbar 25%), pelvis (12%), hip and femur (10%), knee and tibia (10%), ribs (7%), ankle, foot, or shoulder (2%), elbow or wrist (2%), and multiple sites (3%) [1]. Tuberculosis occurring in the small bones of the hand and foot, i.e., metacarpals, metatarsals, and phalanges, is termed tubercular dactylitis or spina ventosa [5]. This clinical entity has a proclivity for smaller bones of the hand than the foot [6]. Spina ventosa is not so common after the age of five, with about 85% of cases reported in children younger than six years [6].

A rare case of a 13-year-old Indian girl is presented who had a painfully swollen left index finger with purulent discharge. Histopathology of the pus samples identified the cause as tuberculous, and she was initiated on treatment per the national guidelines.

## Case presentation

A 13-year-old Muslim, unmarried, non-diabetic girl belonging to a low socioeconomic group came as a referral case from a private set-up with complaints of painful swelling involving her left index finger with purulent discharge for three months. The swelling was insidious in onset over three months and gradually associated with pain, and there was a yellow-colored, non-fowl-smelling, non-blood-tinged, purulent discharge from it for the past 15 days. The pain was initially mild but increased to impact her daily activities. Her pain subsided for a short while after taking an over-the-counter nonsteroidal anti-inflammatory drug.

There was no prior history of weight loss, a cough, a fever, or any other tuberculosis-related constitutional symptoms. She had never smoked and was a student. Additionally, neither she nor any of her acquaintances had a history of tuberculosis. Furthermore, there was no history of trauma, falls, or visits to night shelters or refugee camps. She was sexually inactive and there was no history of child abuse.

A general examination indicated a female with stable hemodynamics. She had a slim build (BMI 18 kg/m²) and showed no signs of pallor, icterus, clubbing, cyanosis, or pretibial edema. Her systemic evaluation revealed nothing noteworthy.

**Figure 1:**
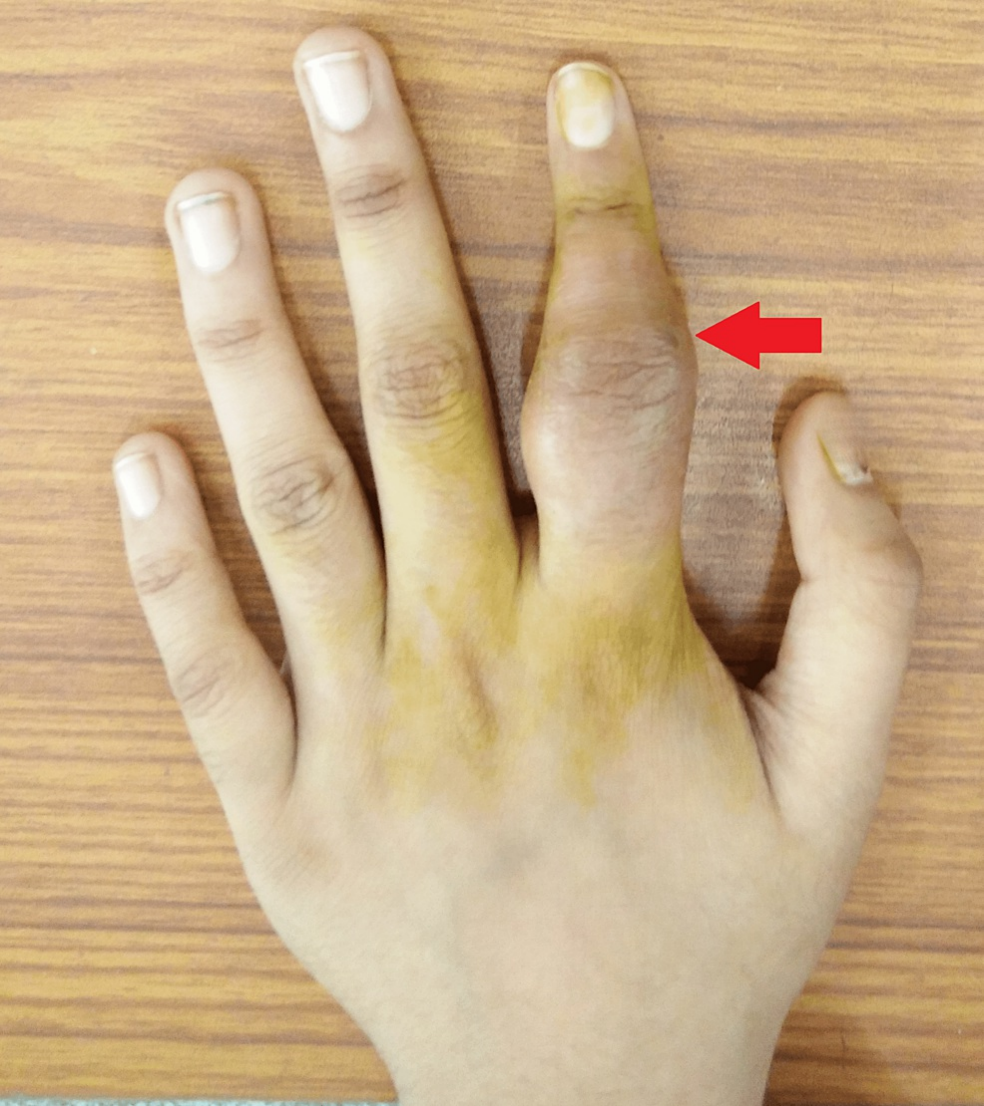
Gross image showing swollen proximal and middle phalanges of the left index finger

On local examination, the proximal and middle phalanges of the left index finger were noticeably enlarged, hard, and fusiform, with erythema in the adjoining skin and no local rise in temperature (Figure 1).

**Figure 2:**
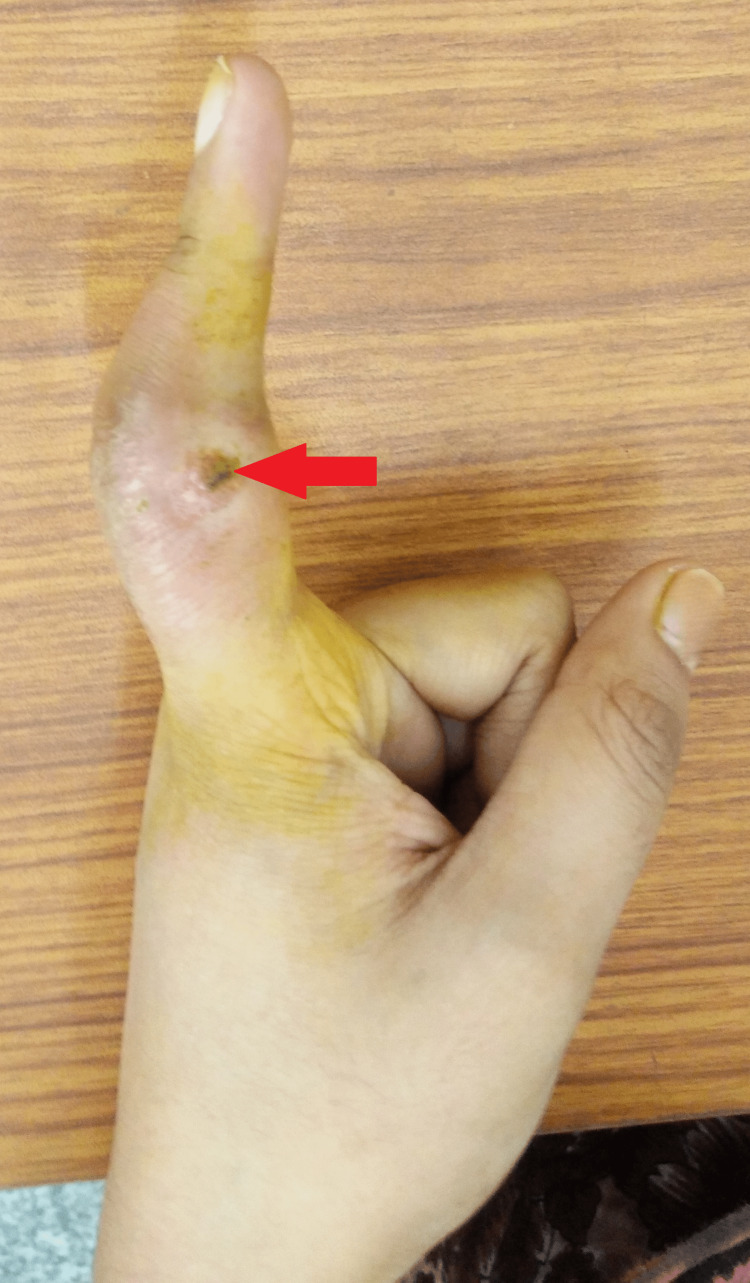
Gross image showing discharging sinus in the left index finger

The proximal and distal interphalangeal joints were stiff with limited and painful movements. There was a discharging sinus about 1 x 1 cm in the medial aspect of the left index finger (Figure 2).

A preliminary diagnosis of chronic pyogenic osteomyelitis was made with a differential diagnosis of fungal dactylitis, syphilitic dactylitis, enchondroma, brucellosis, and tuberculous dactylitis. She was advised to undergo routine serological examinations as well as chest and left-hand radiographs. The results of a chest radiograph for pulmonary tuberculosis were unremarkable.

**Figure 3:**
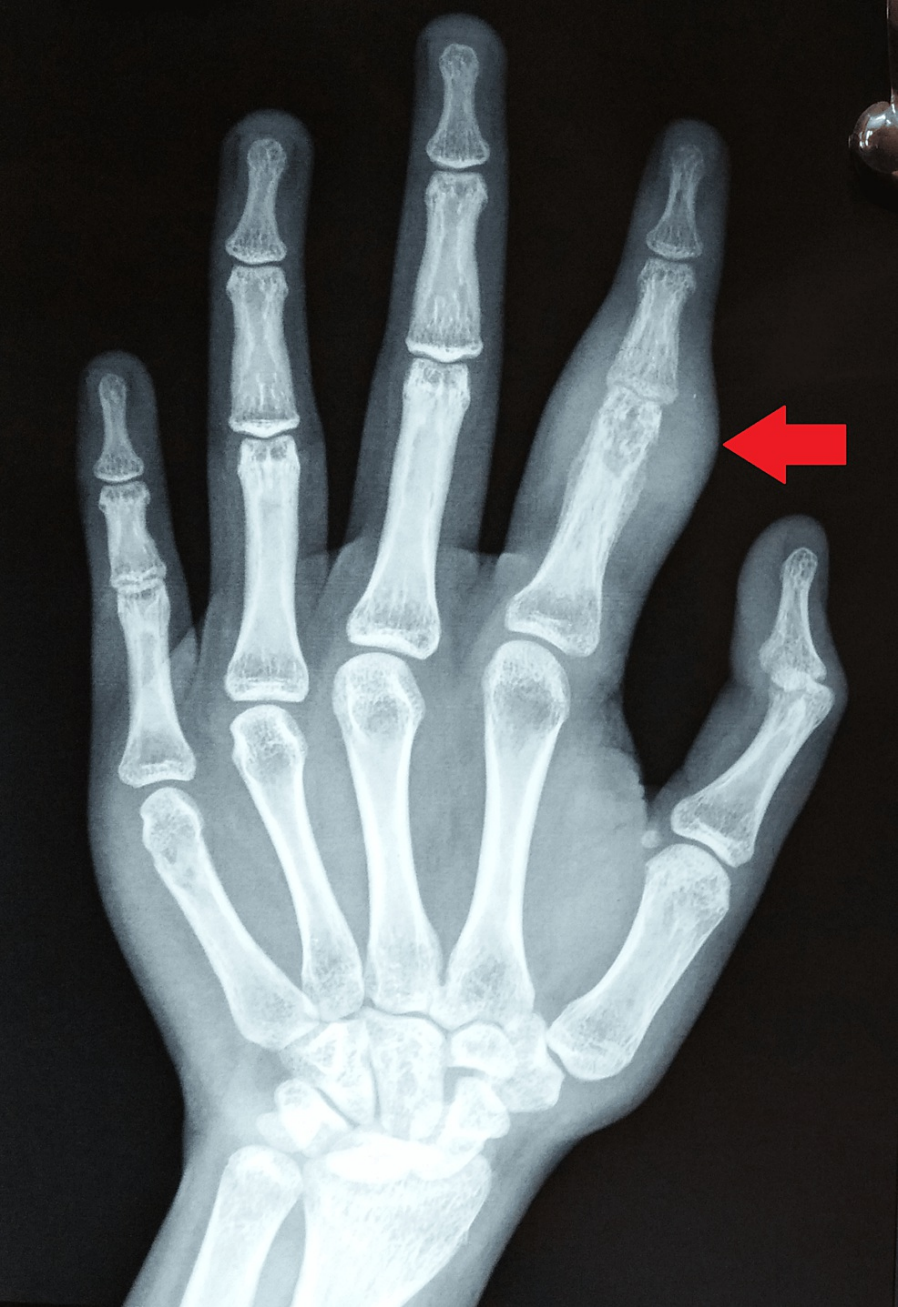
Plain radiographs of the left hand (postero-anterior) view showing soft tissue swelling with cortical erosion in the distal part of the proximal phalanx and a slight periosteal reaction

**Figure 4:**
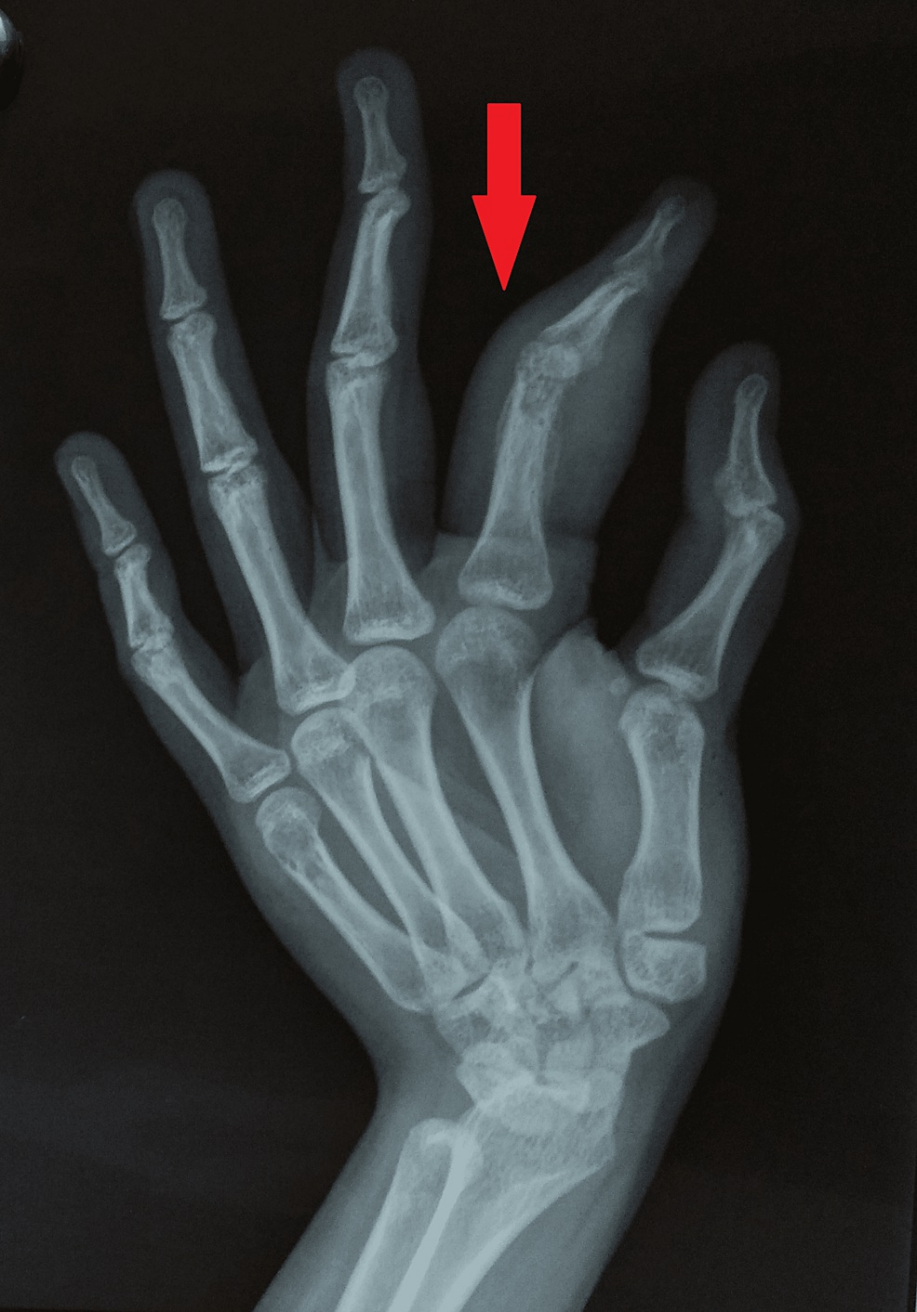
Plain radiograph of the left hand (oblique view) showing cortical erosion in the distal part of the proximal phalanx and soft tissue swelling

Laboratory results suggested a raised erythrocyte sedimentation rate of 46 mm in the first hour with a hemoglobin of 11.1 g/dL. Her HIV, venereal disease research laboratory tests, and hepatitis panel (A, B, and C) were negative. Besides, C-reactive proteins, leukocyte count, absolute lymphocyte and neutrophil count, absolute eosinophil count, and brucella antibody titer results were unremarkable. The rheumatoid factor was negative, but her Mantoux test was strongly positive (30 mm). The plain radiographs of the left hand were suggestive of soft tissue swelling with cortical erosion in the distal part of the proximal phalanx and a slight periosteal reaction giving an impression of spina ventosa (Figures 3-4).

An ultrasound-guided biopsy was performed, and the results revealed Langhans giant cells and granulomatous inflammation affecting the dermis and subcutaneous fat with a necrotic background. Samples for Gram staining and culture for bacteria, fungi, and mycobacteria were negative. Additionally, samples were sent for line-probe assay and cartridge-based nucleic acid amplification testing. Both samples tested positive for *Mycobacterium tuberculosis* (low), and neither had any rifampicin or isoniazid resistance. As a consequence, she was prescribed a 12-month treatment with anti-tubercular chemotherapy after obtaining a final diagnosis of tubercular dactylitis of the distal portion of the proximal phalanx of the left index finger (Table 1).

**Table 1:** Anti-tubercular treatment for 12 months

Phase	Drug	Dose	Duration
Intensive phase	Rifampicin	10 mg/kg	56 days
	Pyrazinamide	25 mg/kg	56 days
	Ethambutol	15 mg/kg	56 days
	Isoniazid	5 mg/kg	56 days
Continuation phase	Rifampicin	10 mg/kg	40 weeks
	Ethambutol	15 mg/kg	40 weeks
	Isoniazid	5 mg/kg	40 weeks

Throughout the entire course of treatment, pyridoxine (1 mg/kg/day) tablets were also advised, and dietary counseling regarding a high-protein diet and treatment adherence was provided. She fared well for the initial two months on the anti-tubercular treatment without any adverse drug reactions with a reduction of her swelling and pain. Her erythrocyte sedimentation rate was 26 mm in the first hour. She requested to be transferred to her native village after the start of the continuation phase; hence, she was transferred out. However, she was urged to have routine follow-ups at the outpatient orthopedic and infectious diseases departments of her hometown but was lost to follow-up.

## Discussion

In impoverished nations, tuberculosis is common [1]. Only 10-15% of cases of tuberculosis are extrapulmonary, making it relatively uncommon [6]. Additionally, only 1-5% of tuberculosis patients have musculoskeletal involvement, which accounts for roughly 10-18% of all cases of extrapulmonary tuberculosis [5,6]. Even in endemic countries, there is a paucity of literature on the tuberculosis of the small bones of the hand [6].

Boyer first recognized spina ventosa, also known as tubercular dactylitis, in 1803, and Nelaton demonstrated the condition's tuberculous genesis in 1837 [7]. It was identified by histological means by Rankin in 1886 and roentgenographically by Feilchenfeld in children in 1896 [6]. Spina ventosa is a general term that refers to any lesion of the bone that causes rising subperiosteal hyperplasia and escalating cortical absorption surrounding the medullary canal until radiological identification of inflated and destructed bone [7]. Between 2% and 4% of all cases of skeletal tuberculosis often exhibit it [6]. The proximal phalanx of the index and middle fingers is the bone most frequently affected [8]. Data on this condition is scarce, primarily because it has a slow progression and only rarely manifests systemically [9].

The illness primarily spreads to the small bones through lymphohematogenous spread [9]. However, this disease may also be related to direct infection post-trauma [10]. The differential diagnosis includes fungal dactylitis, syphilitic dactylitis, enchondroma, brucellosis, and sarcoidosis. All efforts were made in the present case to rule out all the differentials before treatment initiation.

Since the majority of patients with tuberculosis arrive with a late diagnosis with no evidence of active pulmonary tuberculosis in >50% of patients and lack constitutional symptoms, diagnosis is frequently delayed [5,6]. Other contributing reasons include the paucibacillary character of the disease, a lack of knowledge among treating practitioners, clinical symptoms that overlap with those of other musculoskeletal diseases, and vague presentations on multimodal imaging [9]. Soft tissue edema and periostitis are frequently the first symptoms, which progress to expansile bone disintegration and sequestrum development [9].

Anti-tubercular medications are primarily used for management [5,6]. The prognosis is usually good and treatment essentially lasts for 12 months, as recommended by the national guidelines [11]. The patient's status at the end of a year is assessed, which determines the further course of management [11]. Surgical interventions are considered in some cases for diagnosis and treatment (like in carpal tunnel syndrome, drainage of abscess, synovial resection, etc.).

A case similar to ours was presented by Abebe et al. in a four-year-old female child from Ethiopia [12]. However, the present case differs from theirs in the absence of constitutional symptoms of tuberculosis, the presence of a painful swelling, the diagnosis with a line probe assay, and the cartridge-based nucleic acid amplification test of the biopsied samples [12].

Overall, we described a young girl who had an uncommon instance of spina ventosa in her left index finger. In this instance, it is important to emphasize the need for such uncommon clinical presentations to be recorded in the literature since doing so will benefit both the treating physicians and the patients. Particularly in a situation where, even in high-burden countries, cases of spina ventosa are relatively infrequent.

## Conclusions

This unusual case involves a 13-year-old unmarried Indian girl who complained of swelling in her left index finger with pain and a discharging sinus. In this immunocompetent case, a very high index of suspicion was needed to identify and start care because there was no history of trauma or tuberculosis. Anti-tubercular medications were the cornerstone of the 12-month course of treatment after the establishment of diagnosis by histopathology, line probe assay, and cartridge-based nucleic acid amplification test, especially in the absence of pulmonary involvement.
